# The lncRNAMALAT1-WTAP axis: a novel layer of EMT regulation in hypoxic triple-negative breast cancer

**DOI:** 10.1038/s41420-024-02058-4

**Published:** 2024-06-11

**Authors:** Martina Dragonetti, Chiara Turco, Anna Benedetti, Frauke Goeman, Mattia Forcato, Stefano Scalera, Matteo Allegretti, Gabriella Esposito, Francesco Fazi, Giovanni Blandino, Sara Donzelli, Giulia Fontemaggi

**Affiliations:** 1 Translational Oncology Research Unit, IRCCS Regina Elena National Cancer Institute, Via Elio Chianesi 53, 00144 Rome, Italy; 2 SAFU Unit, IRCCS Regina Elena National Cancer Institute, Via Elio Chianesi 53, 00144 Rome, Italy; 3 Department of Life Sciences, University of Modena and Reggio Emilia, Modena, Italy; 4 Biostatistics and Bioinformatics Unit, Clinical Trial Center, IRCCS Regina Elena National Cancer Institute, Rome, Italy; 5 Department of Anatomical, Histological, Forensic & Orthopaedic Sciences, Section of Histology & Medical Embryology, Sapienza University of Rome, Via A. Scarpa, 16, 00161 Rome, Italy

**Keywords:** Breast cancer, Long non-coding RNAs

## Abstract

Early metastatic disease development is one characteristic that defines triple-negative breast cancer (TNBC) as the most aggressive breast cancer (BC) subtype. Numerous studies have identified long non-coding RNAs (lncRNA) as critical players in regulating tumor progression and metastasis formation. Here, we show that MALAT1, a long non-coding RNA known to promote various features of BC malignancy, such as migration and neo angiogenesis, regulates TNBC cell response to hypoxia. By profiling MALAT1-associated transcripts, we discovered that lncRNA MALAT1 interacts with the mRNA encoding WTAP protein, previously reported as a component of the N6-methyladenosine (m6A) modification writer complex. In hypoxic conditions, MALAT1 positively regulates WTAP protein expression, which influences the response to hypoxia by favoring the transcription of the master regulators HIF1α and HIF1β. Furthermore, WTAP stimulates BC cell migratory ability and the expression of N-Cadherin and Vimentin, hallmarks of epithelial-to-mesenchymal transition (EMT). In conclusion, this study highlights the functional axis comprising MALAT1 and WTAP as a novel prognostic marker of TNBC progression and as a potential target for the development of therapeutic approaches for TNBC treatment.

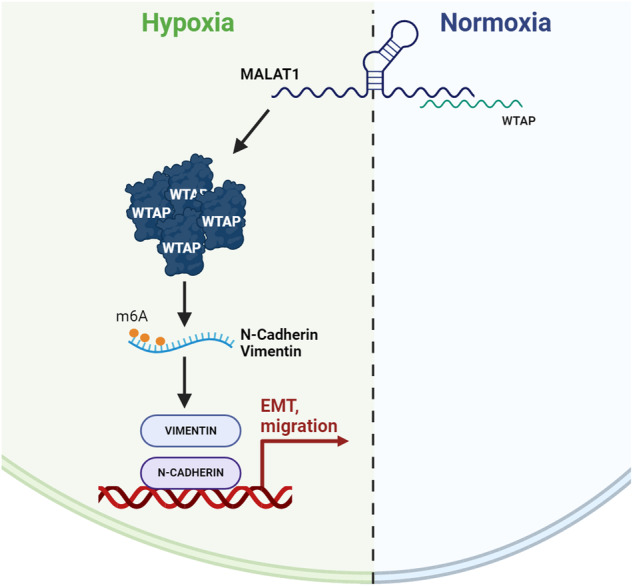

## Introduction

Among BC subtypes, TNBC is the most aggressive, affects young women, and shows a high rate of recurrence/metastasis. About 40% of TNBC experiences recurrence within 5 years after the diagnosis [1]. Due to its molecular characterization, TNBC is the only BC subtype that does not include targeted therapy as a possible treatment [2]. The absence of biomarkers to predict recurrence negatively affects the prevention of tumor relapses. Among the factors affecting TNBC aggressiveness there are tumor cells adaptation to hypoxia [3] and EMT, a functional trait that promote tumor invasiveness. Hypoxia is a common process during tumor growth since the surrounding vasculature becomes inadequate to sustain the high oxygen demand of cancer cells. In order to adapt to hypoxia, BC cells activate the hypoxia-inducible factor 1 α (HIF1α) that translocates to the nucleus and coordinates gene expression changes. Among others, HIF1α activates, either directly or indirectly, most of the EMT factors, making the cells prone to invade and to metastasize [4]. Therefore, deciphering the molecular mechanisms governing TNBC malignancy could be helpful to move a step forward for patients’ prognosis and targeted therapy.

Metastasis-associated lung adenocarcinoma transcript 1 (MALAT1) is a long non-coding RNA highly conserved through evolution and highly expressed in BC, where it is associated with poor overall survival and disease-specific survival, especially in TNBC [5, 6]. Although it is also localized in the cytoplasm, MALAT1 performs its primary function in the nuclear speckles by regulating gene expression and alternative splicing [7]. Therefore, this noncoding RNA, and its methylated form, is able to reshape cells towards a more oncogenic transcriptome [8]. We have previously shown that MALAT1 is also involved in angiogenesis, cooperating with the serine and arginine rich splicing factor 1 (SRSF1), mutant p53, and inhibitor of DNA-binding 4 (ID4) to induce the expression of oncogenic Vascular Endothelial Growth Factor (VEGF) isoforms [9, 10].

m6A modification has been demonstrated to be the most abundant form of post-transcriptional modification of lncRNAs and messenger RNAs (mRNAs) [11], participating in most steps of RNA metabolism, including mRNA transcription, translation, splicing, folding, degradation, and export, representing a new frontier on fine-tuning gene expression at post-transcriptional level in several tumors [12]. Firstly identified in the human urogenital system, Wilms tumor 1 associated protein (WTAP) is a 50 kDa protein, mainly localized in the nuclear speckles [13]. It is known to take part in mRNA splicing regulation processes [14], but its primary role is to be the “adapter” of the m6A writer complex. WTAP does not have methylation activity but interacts with the METTL3–METTL14 complex that mediates m6A deposition on coding and non-coding RNAs [15]. The m6A has been reported to control both tumor-promoting and tumor-suppressor genes. In TNBC, WTAP has been linked to cancer progression, glycolysis, and drug resistance [16, 17]. However, much remains to be done to fully understand its functions in remodeling BC progression [18]. WTAP is widely expressed in a variety of human tissues and its expression is dysregulated in cancer through different mechanisms. Numerous studies have explained the role of different noncoding RNAs (ncRNAs), including microRNAs (miRNAs), lncRNAs, and piwi-interacting RNAs (piRNAs) in tumor development. A number of these ncRNA have been shown to play a role in regulating WTAP expression in several tumor including large B-cell lymphoma, renal carcinoma, hepatocellular carcinoma, osteosarcoma, and others [19, 20].

To investigate if the crosstalk between MALAT1 and molecular components of the m6A function orchestrates gene expression in cancer cells, we evaluated the functional relationship between MALAT1 and WTAP mRNA.

Here, we highlight a new role of MALAT1-WTAP interaction axis in regulating processes that worsen the clinical outcome in TNBC. By binding to WTAP transcript, MALAT1 stabilizes WTAP protein in TNBC cell lines. Under hypoxic condition, WTAP is required for the expression of two major hypoxic markers, HIF1α and HIF1β, underlying the relevant role of MALAT1-WTAP axis in regulating adaptation of the tumor cells to hypoxia, a fundamental step in tumor progression. In addition, we have shown that MALAT1-WTAP interaction is essential in promoting cell migration by affecting the expression of two important markers of epithelial to motile mesenchymal cells transition, N-Cadherin and Vimentin. These results indicate MALAT1 and WTAP as key molecular players of the TNBC progression by facilitating adaptation of the tumor to hypoxia and promoting metastasis.

## Results

### ChIRP-seq analysis highlights WTAP as a MALAT1’s mRNA interactor in triple-negative breast cancer cells

**Fig. 1:**
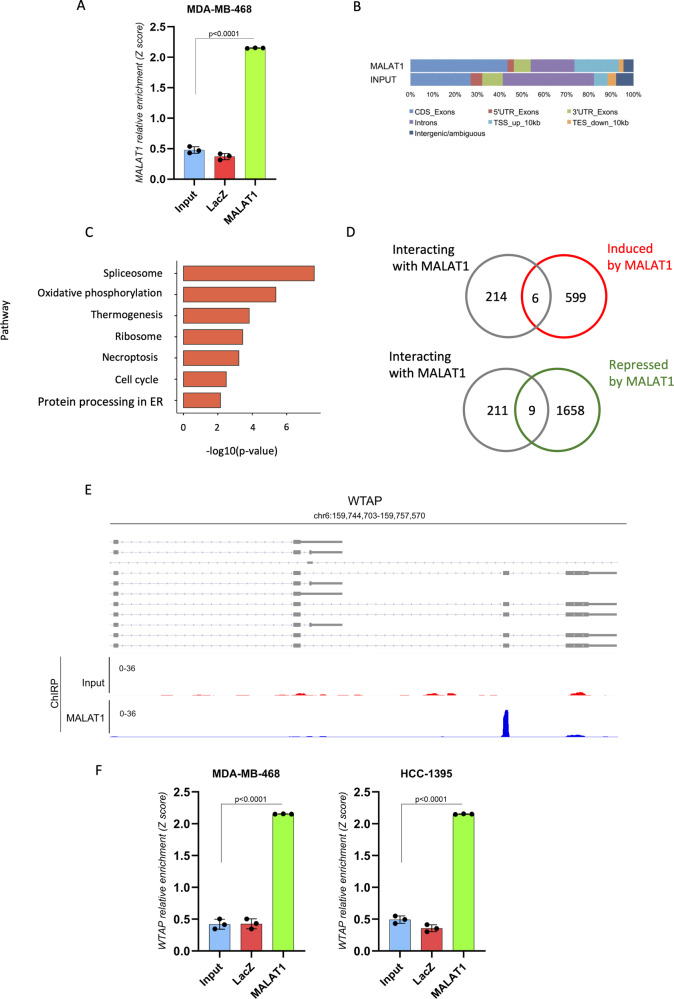
ChIRP-seq analysis highlights WTAP as a MALAT1’s mRNA interactor in triple-negative breast cancer cells. **A** RT-qPCR analysis of lncRNA MALAT1 in ChIRP assay. ChIRP assay was performed to recover MALAT1 lncRNA and its associated RNAs by using a set of biotinylated oligonucleotides complementary to MALAT1 RNA sequence (MALAT1) and a couple of negative control oligonucleotides (LacZ), (*N* = 3 independent biological replicates), *p* value has been calculated by paired, two-tailed Student’s *t*-test. **B** Analysis of the distribution of RNA-seq reads among MALAT1-interacting RNAs recovered through ChIRP assay (MALAT1) and in total RNA sample (INPUT). **C** Pathway enrichment analysis (KEGG Pathways) of MALAT1-interacting genes identified by ChIRP-RNAseq. **D** Venn diagrams showing the intersection between transcripts bound by MALAT1 in ChIRP-RNAseq and mRNAs positively or negatively modulated upon MALAT1 silencing in MDA-MB-468. **E** Representative tracks from ChIRP-RNAseq results for WTAP gene in MDA-MB-468 cells. Tracks from input and MALAT1 samples are shown. **F** RT-qPCR analysis of WTAP expression in ChIRP experiments in MDA-MB-468 (*N* = 3) and HCC1395 (*N* = 3). Relative enrichment over GAPDH expression is shown.

To decipher the molecular mechanisms at the basis of the role exerted by MALAT1 in BC, we performed profiling of MALAT1-interacting mRNAs. To this end, we carried out ChIRP experiments using a panel of 16 antisense oligonucleotides complementary to MALAT1 sequence in MDA-MB-468 cells (Fig. 1A). Oligonucleotides complementary to LacZ mRNA sequence were included as negative control (Fig. 1A). Profiling of MALAT1-associated RNAs was subsequently carried out by ChIRP-seq on the Illumina platform. Analysis of the distribution of ChIRP-seq reads evidenced that MALAT1 mainly interacts with exonic regions (CDS, 5′-UTR and 3′-UTR) (nearly 55% of the reads) (Fig. 1B). We identified 220 MALAT1-interacting RNAs (FC MALAT/Input > 5; *p* < 0.05), functionally involved in various biological pathways, included the previously reported “Ribosome”, “Spliceosome” and “Oxidative phosphorylation” (Fig. 1C). Intersection between the identified MALAT1-interacting mRNAs and a list of mRNAs modulated after 48 h of MALAT1 silencing in MDA-MB-468, identified by a microarray assay, revealed very low overlapping mRNAs (Fig. 1D). This suggests that MALAT1-interacting mRNAs might be regulated by MALAT1 mainly at post-transcriptional level. The ChIRP-seq analysis revealed that MALAT1 associates with the exon region of the regulatory subunit of m6A methyltransferase complex, WTAP. The enrichment of MALAT1 on WTAP mRNA in ChIRP-seq was evaluated by comparing the MALAT1 vs Input samples (FC = 7.06; pval = 2.80E-18; qval = 7.14E-16). Visualization of reads’ distribution along WTAP gene allowed identifying exons 6–7–8 of WTAP as the most MALAT1-enriched (Fig. 1E). We therefore drew PCR primers on these exonic regions for validation experiments. Independent biological replicates of ChIRP experiments for MALAT1 were carried out in MDA-MB-468 and HCC1395 cell lines. As shown in Fig. 1F, evaluation of ChIRP experiments by real-time PCR showed that WTAP mRNA was enriched in the RNA recovered by using MALAT1-complementary oligonucleotides compared to Input samples in both cell lines.

### MALAT1 regulates WTAP expression in hypoxic triple-negative breast cancer cells

**Fig. 2:**
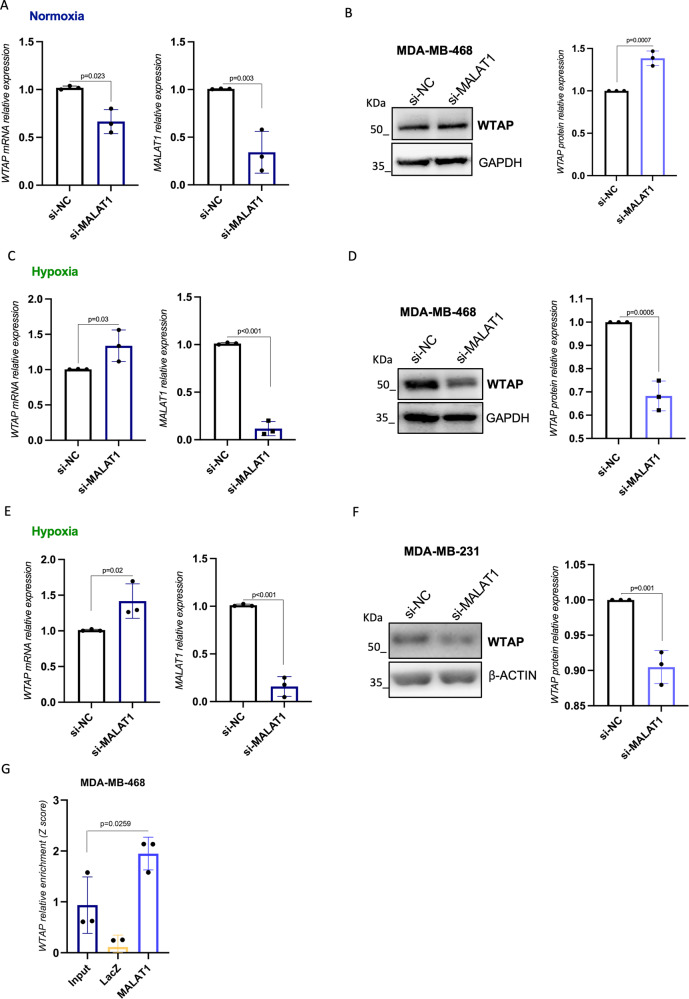
MALAT1 regulates WTAP expression in hypoxic breast cancer cells. **A** RT-qPCR analysis of respectively MALAT1 and WTAP in MDA-MB-468 cells grown in normoxia transfected with a siRNA to silence MALAT1 (si-MALAT1) or control siRNA (si-NC) for 72 h. (*N* = 3 independent biological replicates). *p* value has been calculated by unpaired, two-tailed Student’s *t-*test. **B** Western blot analysis of WTAP in MDA-MB-468 cells transfected with siRNA directed to MALAT1 (si-MALAT1) or control siRNA (si-NC) for 72 h. GAPDH staining was used as loading control. **C**–**E** RT-qPCR analysis of WTAP mRNA and MALAT1 lncRNA after transfection of siRNA to silence MALAT1 (si-MALAT1) or control siRNA (si-NC) in MDA-MB-468 and MDA-MB-231 cells grown in hypoxia. (*N* = 3 independent biological replicates). **D**–**F** Representative Western blot analysis of WTAP protein in MDA-MB-468 and MDA-MB-231 treated as in (**C**–**E**) with the relative quantification (right graphs). GAPDH and β-ACTIN staining were used as loading control. **G** RT-qPCR analysis of WTAP expression in ChIRP experiments in hypoxic MDA-MB-468 (*N* = 3). Relative enrichment over GAPDH expression is shown.

Aimed at evaluating if MALAT1-WTAP mRNA interaction impacts on the expression of WTAP, we silenced the expression of MALAT1 by siRNA transfection in MDA-MB-468 cells and evaluated the mRNA and protein expression of WTAP. As shown in Fig. 2A, interference of MALAT1 caused a slight but significant downregulation of WTAP mRNA, as assessed through RT-qPCR. However, analysis by western blot of WTAP protein showed no great modulation upon MALAT1 depletion (Fig. 2B, Supplementary Fig. 3A). As mounting evidence is showing specific functions of MALAT1 in hypoxic conditions [21], we next evaluated whether silencing of MALAT1 modulates the expression of WTAP in hypoxic cells. To this end, MDA-MB-468 and MDA-MB-231 cells were transfected with siRNA for MALAT1 for 24 h and moved to hypoxia for further 48 h. Analysis of WTAP expression showed that, while WTAP mRNA was upregulated upon MALAT1 silencing (Fig. 2C–E), WTAP protein was significantly downregulated after depletion of MALAT1 in hypoxia in both cell lines (Fig. 2D–F, Supplementary Fig. 3B, C). Immunofluorescence analysis further confirmed our observation (Supplementary Fig. 1A). ChIRP assay verified the interaction of MALAT1 with WTAP mRNA also in MDA-MB-468 under hypoxia condition (Fig. 2G).

All together these findings suggest that MALAT1 exerts a positive regulatory activity of WTAP at the post-transcriptional level under hypoxic condition.

To explore the molecular mechanism through which MALAT1 regulates WTAP protein expression, we performed RIP experiments targeting eIF4B, a translation initiation factor crucial for mRNA binding to ribosomes, upon MALAT1 silencing under hypoxic conditions. As shown in Supplementary Fig. 1B, we observed a reduction in eIF4B binding to WTAP mRNA upon MALAT1 depletion. The obtained results reveal an involvement of MALAT1 in WTAP translation.

### WTAP mRNA and protein are differently modulated in tumor vs normal tissues with different clinical outcome

**Fig. 3:**
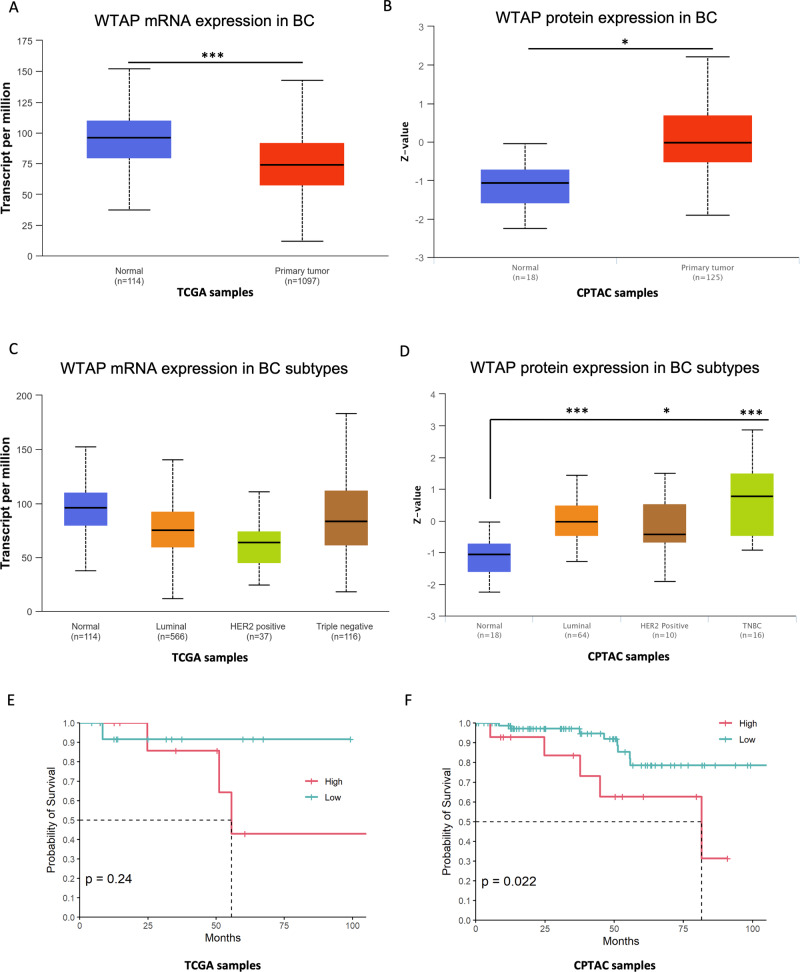
WTAP mRNA and protein are differently modulated in tumor vs normal tissues with different overall survival. **A**, **B** Box plot showing the expression of WTAP mRNA and protein in TCGA and CPTAC datasets of Breast invasive carcinoma *p < 0.05; ***p < 0.0005. **C**, **D** Box plot showing the expression of WTAP mRNA and protein in TCGA and CPTAC datasets of BC subtypes *p < 0.05; ***p < 0.0005. **E** Kaplan–Meier analysis showing probability of survival between the two groups: high WTAP mRNA expression (*N* = 10 patients) and low WTAP mRNA expression (*N* = 14 patients) in TNBC patients (*p* = 0.24). **F** Kaplan–Meier analysis showing probability of survival between the two groups: high WTAP protein expression (*N* = 14 patients) and low WTAP protein expression (*N* = 84 patients) in TNBC patients (*p* = 0.022).

Consistent with the discordant expression profiles of WTAP mRNA and protein observed in breast cancer cell lines, we next evaluated WTAP expression in cancer datasets. To this end we interrogated the UALCAN platform (The University of ALabama at Birmingham CANcer data analysis portal) (http://ualcan.path.uab.edu/index.html), a web resource for analyzing cancer OMICS data. The analysis showed that WTAP mRNA and protein are, respectively, downregulated and upregulated in breast cancer vs normal tissues (Fig. 3A, B). This result supports our observation toward the contradictory behavior of WTAP mRNA and protein in breast cancer cell lines. Pan Cancer analysis of WTAP revealed a similar trend in other cancer types, for example in Lung Adenocarcinoma (Supplementary Fig. 1C). The evaluation of WTAP mRNA expression within the different BC subtypes reported a non-significative difference among them (Fig. 3C), while WTAP protein resulted significantly upregulated in all the subtypes compared to normal tissue, especially in TNBC (Fig. 3D). Our analysis also highlighted that while WTAP mRNA was not able to discriminate for a difference in the overall survival (*p* = 0.24) (Fig. 3E), WTAP protein expression significantly correlated with a worst overall survival (*p* = 0.022) (Fig. 3F). High WTAP group also showed a worst disease-free survival (DFS) (Supplementary Fig. 1D). These findings highlight a clinical relevance of WTAP in breast cancer.

### WTAP sustains HIF1α in hypoxic TNBC cells

**Fig. 4:**
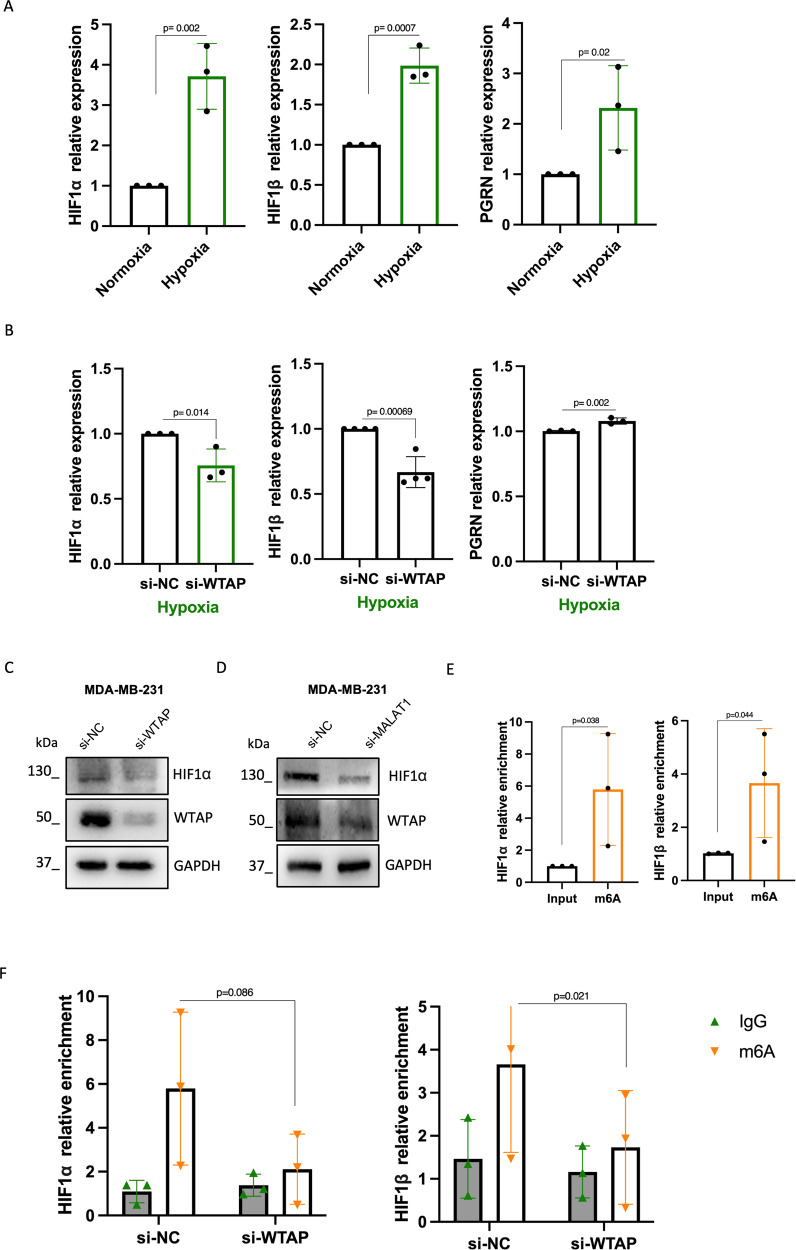
WTAP sustains HIF1α in hypoxic TNBC cells. **A** RT-qPCR analysis of respectively HIF1α, HIF1β and PGRN in MDA-MB-231 cells grown in normoxia and hypoxia for 48 h (*N* = 3 independent biological replicates). *p* Value has been calculated by unpaired, two-tailed Student’s *t*-test. **B** RT-qPCR analysis of HIF1α, HIF1β and PGRN mRNA after transfection of siRNA to silence WTAP (si-WTAP) or control siRNA (si-NC) in MDA-MB-231 cells grown in hypoxia (*N* = 3 independent biological replicates). **C**, **D** Western blot analysis of HIF1α and WTAP in MDA-MB-231 cells transfected with siRNA directed to WTAP (si-WTAP) (left) and MALAT1 (si-MALAT1) (right) or control siRNA (si-NC) for 72 h (in hypoxia). GAPDH staining was used as loading control. **E** RT-qPCR analysis of HIF1α and HIF1β showing relative enrichment after m6A immunoprecipitation of hypoxic MDA-MB-231. **F** RT-qPCR analysis of HIF1α and HIF1β in m6A immunoprecipitation. m6A IP was performed using Ab against m6A modification in MDA-MB-231 upon silencing of WTAP (si-WTAP) or control siRNA (si-NC) in hypoxia. IgG were used as control.

In light of the marked effect of MALAT1 silencing on WTAP protein expression in hypoxic condition, we aimed at understanding how WTAP impacts on hypoxia. Expression analysis of the two major hypoxic markers, HIF1α and HIF1β, by real-time PCR in TNBC cells, showed a strong induction of these two factors in hypoxia respect to normoxia, as expected (Fig. 4A). The cofactor Progranulin was also significantly upregulated (Fig. 4A). Interestingly, in hypoxic conditions and following WTAP depletion, we observed a downregulation of HIF1α and HIF1β mRNA levels compared to control cells (Fig. 4B). As assessed by Western Blot, also HIF1α protein was significantly downregulated upon WTAP silencing in hypoxic MDA-MB-231 (Fig. 4C, Supplementary Fig. 4A). Coherently with MALAT1 impact on WTAP protein, cells undergoing MALAT1 interference showed an HIF1α reduced protein level (Fig. 4D, Supplementary Fig. 4B). Given that the most well-documented function of WTAP is the deposition of m6A methylation on transcripts, leading to their stabilization, we investigated whether WTAP affects the methylation status of HIF1α and HIF1β transcripts through m6A immunoprecipitation (m6A IP) by precipitating methylated RNA using an anti-m6A antibody. As shown in Fig. 4E, we initially observed a significant methylation of HIF1α and HIF1β transcripts under hypoxic conditions. Subsequently, upon WTAP silencing, we observed a significant decrease in the methylation levels of HIF1α and HIF1β (Fig. 4F).

These results indicated a role of WTAP in the regulation of cell response to hypoxia.

### WTAP regulates TNBC cell migration in hypoxia

**Fig. 5:**
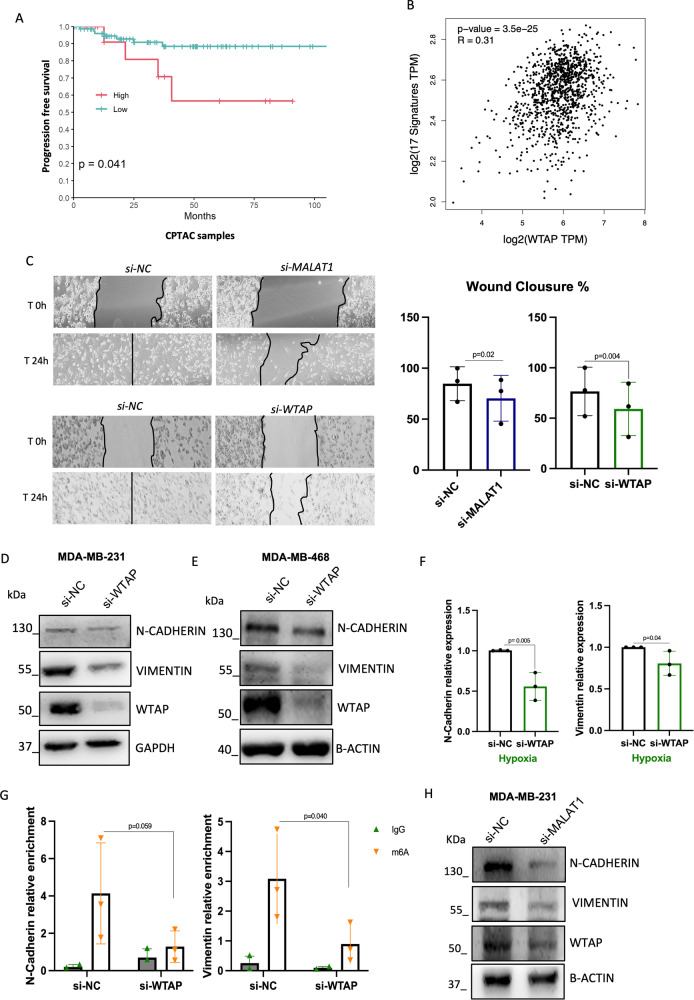
WTAP regulates TNBC cell migration in hypoxia. **A** Kaplan–Meier showing progression-free survival (PFS) between the two groups: high WTAP protein expression (*N* = 14 patients) and low WTAP protein expression (*N* = 84) in TNBC patients (*p* = 0.041). **B** Scatter Plot analysis from GEPIA2 webtool showing a correlation between WTAP and a signature of 17 EMT genes (*p* = 3.5e25). **C** Wound-healing migration assay from MDA-MB-231 cells transfected with siRNA directed to both MALAT1 (si-MALAT1) and WTAP (si-WTAP) or control siRNA (si-NC) for 24 h in hypoxia. The wound closure percentage is represented in the right graphs. p-value has been calculated by unpaired, two-tailed Student’s *t*-test. **D**, **E** Representative Western blot analysis of N-Cadherin, Vimentin and WTAP proteins in MDA-MB-468 and MDA-MB-231 after transfection of siRNA to silence WTAP (si-WTAP) or control siRNA (si-NC) for 72 h (48 h of which in hypoxia) with the relative quantification (right graphs). GAPDH and β-ACTIN staining were used as loading control. **F** RT-qPCR analysis of N-Cadherin and Vimentin mRNA treated as in (**D**, **E**) (*N* = 3 independent biological replicates). **G** RT-qPCR analysis of N-Cadherin and Vimentin in m6A immunoprecipitation. m6A IP was performed using Ab against m6A modification in MDA-MB-231 upon silencing of WTAP (si-WTAP) or control siRNA (si-NC) in hypoxia. IgG were used as control. **H** Western blot analysis of N-Cadherin, Vimentin and WTAP in MDA-MB-231 cells transfected with siRNA directed to MALAT1 (si-MALAT1) or control siRNA (si-NC) for 72 h (48 h of hypoxia). β-ACTIN staining was used as a loading control.

Our previous analysis on clinical outcomes also revealed an association between high WTAP protein and an unfavorable progression free survival (PFS) (Fig. 5A). Following analysis from GEPIA2 webtool, we observed a positive and strong correlation between WTAP and a signature of 17 EMT genes, listed in Supplementary Table 1 (Fig. 5B). Considering the role of WTAP in tumor progression, we decided to perform a wound-healing migration assay upon silencing of WTAP and MALAT1 in hypoxic condition. In both cases, the ability of the cells to migrate was significantly affected (Fig. 5C). CSFE assay and PI staining were carried out to confirm that proliferation and cell death were not altered in the same conditions (Supplementary Fig. 2A–B). Therefore, we analyzed two important mesenchymal markers, N-Cadherin and Vimentin, in our experimental settings. These key factors of the transition from epithelial to motile mesenchymal cells, resulted significantly downregulated upon silencing of WTAP after 48 h of hypoxia in MDA-MB-231 (Fig. 5D, Supplementary Fig. 5A) and in MDA-MB-468 (Fig. 5E, Supplementary Fig. 5B). Likewise, N-cadherin and Vimentin mRNAs were significantly downregulated in hypoxic MDA-MB-231 (Fig. 5F).

Based on the pivotal role of WTAP in m6A methylation deposition, we hypothesized that the downregulation of N-Cadherin and Vimentin transcripts was due to an alteration of their methylation status following WTAP interference. Performing an m6A IP, we confirmed that both the transcripts were methylated in hypoxic conditions (Supplementary Fig. 2C–F) and next, we observed a downregulation of the methylation levels on both N-Cadherin and Vimentin mRNAs upon WTAP interference in hypoxia (Fig. 5G).

Due to the demonstrated regulatory effect of WTAP on HIF1α and HIF1β, coupled with the well-documented impact of HIF1α and HIF1β on EMT [4], we aimed to determine whether WTAP’s effect on EMT regulators was HIF1α and HIF1β dependent. To address this, we performed a m6A IP under normoxic conditions. As shown in Supplementary Fig. 2D, we observed a significant reduction in m6A modification on the Vimentin transcript and a notable, albeit not statistically significant, decrease in m6A modification on the N-Cadherin transcript (Supplementary Fig. 2G). Furthermore, we noted a significant downregulation of both Vimentin and N- Cadherin transcripts in normoxia upon WTAP depletion (Supplementary Fig. 2E–H).

These results indicate that the m6A modification, mediated by WTAP, stabilizes Vimentin and N-Cadherin transcripts, leading to the maintenance of their proteins in hypoxic conditions, in a HIF1α/β independent manner.

To strengthen the evidence supporting the MALAT1-WTAP axis, we assessed the expression levels of N-Cadherin, Vimentin, and HIF1α/β proteins in MDA-MB-231 cells following MALAT1 depletion (Fig. 5H, Supplementary Fig. 5C). Our findings revealed a notable reduction in the expression of these proteins, further supporting the involvement of the MALAT1-WTAP axis in regulating their expression.

## Discussion

LncRNAs are one of the leading actors of tumorigenesis and metastasis in breast cancer. The abundant lncRNA MALAT1 has been associated with a poorly differentiated and aggressive phenotype of mammary carcinomas [22]. Here we have shown that lncRNA MALAT1 is functionally associated to the m6A adapter protein WTAP. Screening for MALAT1 interactions, by ChIRP-seq experiments, we have found that MALAT1 interacts with the exonic regions (exons 6–7–8) of WTAP. MALAT1 positively regulates WTAP protein expression, and not its mRNA, specifically in BC cell lines grown in low oxygen concentration, while no decrease was observed when cells were grown in normoxia. Our results highlight the discordant expression profiles of WTAP mRNA and protein not only in breast cancer cell lines, but also extended to cancers datasets. This observation suggests, together with our experiments, for WTAP a post-transcriptional regulation level. Multiple could be the mechanisms through which WTAP protein is maintained at a high level by MALAT1 in hypoxia: for instance, an increasing of the translational rate, due in turn to a stronger interaction with the translation initiation factors [23]. This hypothesis is supported by our observation of reduced binding between eIF4B and WTAP transcript upon MALAT1 depletion under hypoxic conditions.

It is well established that primary tumors and metastases are in a state of hypoxia [24]. Intra-tumoral hypoxia also influences cell proliferation, angiogenesis, metabolism and progression. Of note, numerous lncRNAs are regulated by a hypoxic microenvironment, influencing their interaction with different proteins, leading to different outcomes [25]. Since our evidence suggest that the functional link between MALAT1 and WTAP acts in hypoxia, we looked at the master regulators of hypoxia, HIF1α and HIF1β, whom mRNA levels occurred to be significantly downregulated in TNBC cells, following WTAP depletion.

Hypoxic cells are more aggressive and more prone to migrate, since they activate several oncogenic pathways such that of TGFb, AKT, NFKB and EMT factors, conferring them a specific ability to metastasize [26]. It has recently emerged that WTAP regulates the Hepato Cellular Carcinoma (HCC) progression by controlling the EMT [27]. During EMT, tumor cells loss of epithelial integrity, and gain of mesenchymal traits, lose their junctions, reorganize their cytoskeleton, undergo a change in the signaling programs that define cell shape and gene expression increasing their motility and invasive phenotype. In agreement with the literature, our bioinformatic analysis predicted the existence of a strong correlation between WTAP expression and a signature of 17 EMT genes in BC. Performing wound-healing migration assay upon silencing of WTAP and MALAT1 in hypoxic condition, we observed a significant reduction of the ability of the cells to migrate.

At the functional level, we provide evidence that in hypoxic conditions, WTAP stimulates the m6A methylation of N-Cadherin and Vimentin, two hallmarks of EMT, thus stabilizing their expression and increasing BC cell migratory ability, a condition that could be reverse by silencing WTAP.

According to the role of MALAT1 in worsening tumor progression, we have shown that WTAP expression levels significantly correlate with the worst clinical outcome shortening the overall survival and disease-free survival, identifying WTAP as a new effector of MALAT1.

To date, there are no WTAP inhibitors available for human therapy. It is known that chemical inhibition of METTL3, the catalytic part of the m6A writer complex, enhance the sensitivity of standard target therapy in TNBC [28]. Therefore, it is essential to identify new molecules to target WTAP or its molecular partners to counteract tumor progression [29]. Notably, our study identifies the MALAT1-WTAP axis as a novel player in breast cancer progression and metastasis, highlighting its potential as a tumor biomarker.

## Materials and methods

### Cell culture and transfection

MDA-MB-231 (ATCC® HTB-26, ATCC, Manassas, Virginia, USA) and HCC1395 (ATCC® SC-CRL-2324) cell lines were maintained in RPMI 1640 medium (31870, Thermo Fisher Gibco, Waltham, MA, USA) containing 10% heat-inactivated fetal bovine serum (10270, Thermo Fisher, Gibco) and penicillin/ streptomycin. MDA-MB-468 (ATCC® HTB-132) were maintained in DMEM high glucose (ECB7501L, Euroclone, Milan, Italy) containing 10% FBS, l-Glutamine and penicillin/streptomycin. All cell lines were grown at 37 °C, 5% CO_2_. For transfections, siRNAs (synthesized by IdT, Tema Ricerca, Bologna, Italy) (300 pmol) were transfected with RNAiMax reagent (Thermo Fisher Scientific, Gibco) using 3 × 10^5^ cells in 2.5 ml of medium for 48 h, unless otherwise stated, following manufacturer’s instructions. The sequences of siRNAs used are listed in Supplementary Table 2.

### Hypoxia treatment

To induce hypoxia, cells were infused in a sealed modular incubator chamber (Thermo Fisher Scientific, Rochester, NY, USA) with humidified hypoxic air containing 5% O_2_, 10% CO_2_, 85% N_2_ for 4 min (20 L/min with a pressure of 8–10 Psi ->0.55–0.68 Bar). Subsequently, such chamber was kept in the incubator at 37 °C for 48 h. Control cells were incubated under normoxic conditions (21% O_2_, 5% CO_2_, 37 °C) for equivalent periods.

### RNA isolation and real-time PCR

Total RNA was isolated with TRIzol RNA isolation reagent (Thermo Fisher Scientific, Gibco) and its concentration was measured using a NanoDrop 2000 (Nanodrop Technologies, Wilmington, DE, USA). The integrity of RNA samples (RIN) and quantification of ribosomal RNA peaks (18S and 28S) were assessed using Agilent 2100 Bioanalyzer (RNA 600 nano kit, Agilent Technologies, Santa Clara, SC, USA). Reverse transcription was performed with SuperScript IV VILO Master Mix (Invitrogen, Thermo Fisher Scientific), following manufacturer’s instructions (at 65 °C).

Real-time PCR was carried out on QuantStudio5 Fast Sequence Detection Systems (Applied Biosystems, Carlsbad, CA, USA). All primers were purchased from IdT (Integrated DNA Technologies) if not specified differently. The primers used are listed in Supplementary Table 3. The expression values were calculated by ΔΔCt method and normalized to GAPDH, RNU2, RPL19 or the indicated control genes (Supplementary Table 3).

### Western blot

For the western blot analysis, cells were lysed in NP40 buffer with Tris–HCl pH 7.5; 1 M -NaCl 5 M; EDTA 0.5 M; EGTA 0.5 M; NP40 10% and fresh protease inhibitors. Extracts were sonicated for 10 + 15 s at 80% amplitude and centrifuged at ~12,000 rpm for 10 min to remove cell debris. The protein concentration was measured using a BCA protein assay kit (Thermo Scientific). The lysate was mixed with 4× Laemmli buffer and boiled for 5 min. Total protein extracts were resolved on polyacrylamide gel and then transferred onto nitrocellulose membrane. The following primary antibodies were used: 60188–1-Ig (anti-WTAP, Proteintech, Manchester, UK), SC-47778 (anti-β actin, Santa Cruz, Dallas, TX, USA), SC-47724 (anti-GAPDH, Santa Cruz), SC-7939 (anti-N-Cadherin, Santa Cruz), SC-66001(anti-Vimentin, Santa Cruz), A300-286A (anti-HIF1α, Bethyl, Waltham, MA,USA).

A secondary antibody fused with horseradish peroxidase was used for chemiluminescence detection on a UVITEC instrument (Alliance 4.7 by Uvitec, Cambridge, UK). Proteins quantification was carried out by the tool “Quantification” supplied by Uvitec.

### Immunofluorescence

For immunofluorescence cells were cultured on a cover glass and fixed in 4% formaldehyde PBS, then permeabilized for 10 min with 0.1% Triton X-100 in PBS and blocked for 30 min with BSA 4% in PBS. Incubation with primary antibody for 60188–1-Ig (anti-WTAP, Proteintech) was performed overnight at 4 °C in a humidified chamber in BSA 1% in PBS. Incubation with secondary antibodies was performed for 1 h at RT in a humidified chamber in BSA 1% in PBS with Goat anti-mouse Alexa Fluor 488 (A-11001, Thermo Fisher). Nuclei were stained with Hoechst (33342, Thermo Fisher) and the slides were mounted with glycerol mounting medium. Images were acquired under a Zeiss LSM 900 confocal microscope (Zeiss, Oberkochen, Germany), and images were analyzed with ImageJ/Fiji® software.

### ChIRP assay, ChIRP-seq and bioinformatic analysis

ChIRP was performed as described in Chu et al. [30] 20 × 10^6^ cells were used for each condition. Cells were crosslinked with 1% glutaraldehyde diluted in room temperature PBS. The cross-linking reaction was quenched with 1/10th volume of 1.25 M glycine at room temperature for 5 min. After this, crosslinked cells were first lysate and then sonicated in a Bioruptor (Biosense, Milan, Italy) in a 4 °C water bath at the highest setting with 30 s ON, 45 s OFF pulse intervals. Biotinylated DNA probes were hybridized to RNA in a Hybridization Buffer and incubated at 37 °C for 4 h with shaking. Biotinylated oligonucleotides were recovered using “Dynabeads” MyOne Streptavidin C1 (Invitrogen). After the washing steps, the Proteinease K (Qiagen, Milan, Italy), and the reverse cross-linking, RNA was ready for purification and DNAse (DNAse kit, Invitrogen) treatment, as described previously. The oligonucleotides used for MALAT1 immunoprecipitation are listed in Supplementary Table 4. Lac_Z was used as control.

ChIRP-seq was performed on DNase-treated RNA recovered from pull-down experiments, and from input samples as well. The RNA from the pull-down experiments was controlled on a Bioanalyzer with the Agilent RNA 6000 Pico Kit (Agilent Technologies) to verify the suitable size distribution and to simultaneously quantify the low RNA amounts. The RNA libraries for the RNA sequencing were prepared using the SMARTer Stranded Total RNA Sample Prep Kit-Low Input Mammalian (Takara Bio, USA, Inc). Each ChIRP-seq was conducted combining two independent biological replicates. For the input we used 100 ng total RNA and performed ribosomal depletion with RiboGone (Takara Bio) prior to cDNA and library preparation. For the RNA deriving from the ChIRP experiments, we used 600 pg for cDNA and library preparation following the manufacturer’s instructions. The quality of the resulting libraries was controlled on a Bioanalyzer using the High Sensitivity DNA Kit (Agilent Technologies). The quantification of the libraries was performed by qPCR. Sequencing was carried out on a NextSeq 500 instrument (Illumina Inc., San Diego, CA, USA), sequencing in paired-end mode 76 bp. Paired-end reads were aligned using STAR version 2.7.3a to build version hg38 of the human genome. Reads aligning on ribosomal RNA were discarded using RSeQC. Strand specific coverage files (bigwig) were generated from the aligned reads using deepTools. Counts for GENCODE (version 35) annotated genes were calculated from the aligned reads using featureCounts function of the Rsubread R package and R (version 3.6.3). For the analysis at gene level, counts for MALAT1 were removed, then normalization and differential expression analysis were carried out using edgeR R package. Raw counts were normalized to obtain Counts Per Million mapped reads (CPM) and Fragments Per Kilobase per Million mapped reads (FPKM). Only genes with a CPM greater than 1 in at least one sample were retained for downstream analysis. After removal of reads relative to rRNA we observed enrichment of 220 transcripts (FPKM (MALAT1) > 15; FPKM (input) > 5 FC > 5; *p* value < 0.05).

### Wound-healing assay

To study the coordinated movement of a cell population, a wound-healing assay was used. Cells were plated in a 24-well cell culture plate to form confluent monolayers. After 24 h of culture, a pipette tip was used to create a scratch in each well. After the scratch, cell culture was rinsed twice with phosphate-buffered saline (PBS) 1×, then medium was replaced with serum-free medium. A horizontal reference line on the bottom of each well was made to have a grid for alignment and obtain the same field for each image acquisition. The images of the gap were acquired at 0 h (immediately after scratching) and after 24 h of incubation at 37 °C with 5% CO_2_. The scratch area was quantified using the open sourceopen-source ImageJ/Fiji®, calculating the percentage of wound closure.

### CFSE staining

Proliferation has been assessed by using the CFSE (Thermo Fisher Scientific). Briefly, cells were seeded at a density of 2.5 × 10^5^, transfected with different siRNAs (see above), and monitored up to 48 h. Cells were stained with 5 μM CFSE for 20 min at 37 °C. The dynamic range of the analysis was fixed by comparing the maximum emission of CFSE (assessed after 24 h of culture) and cell autofluorescence. Changes between conditions were evaluated with merge function by the FlowJo v.10 software (Becton &Dickinson, NJ, USA).

### RNA immunoprecipitation (RIP) on lysates cells

Cells were crosslinked with 254-nm UV light 800 mJ/cm2 (using 10-cm dish with 2.5 ml PBS) or with formaldehyde (F.A.) solution (50 mM HEPES-KOH pH 7.5, 100 mM NaCl, 1 mM EDTA, 0.5 mM EGTA, 11% formaldehyde, ddH_2_O) 1% final concentration, 10 min, RT. Cells were resuspended in lysis buffer (Tris–HCl pH 7.5 50 mM, EDTA 1 mM, SDS 0.5%, DTT 1 mM), using 200 µL for each planned immunoprecipitated (IP) sample, and sonicated to obtain a smear not higher than 500 bp. Lysate was treated with DNase (DNAfree, Ambion) and diluted with 400 µL of correction buffer (NP-40, 0.625%, DOC, 0.312%, MgCl2, 5.6 mM, Tris–HCl pH 7.5, 47.5 mM, NaCl, 187.5 mM, glycerol, 12.5%, DTT 1 mM). IP was carried out overnight at +4 °C. For eIF4B IP, were employed the following ChIP-grade antibody from Cell Signaling: #3592. After immunoprecipitation, beads were washed four times with 1 ml of ice-cold NT2 buffer and then resuspended in 100 µL of NET buffer plus 100 µL of proteinase K buffer (200 mM Tris–HCl pH 7.5, 20 mM EDTA pH 8.0, and 100 mM NaCl, 2% SDS). Samples were incubated with 30 ug of proteinase K for 30 min at 55 °C, crosslinking was reversed by incubation at 70 °C for 30 min and RNA was recovered by TRIzol extraction.

### RIP-m6A assay

The method used for m6A- RIP was adapted from Domissini et al. [31] and Molinie et al. [32]. Fragmentation was not performed because it is propaedeutically to sequencing. Isolated RNA (120γ) was incubated with m6A-specific antibody (#ab151230, Abcam, MA, USA) or with control rabbit IgG (#2729, Cell Signaling Technology, MA, USA) antibody for 2 h at 4 °C with rotation. The immunoprecipitation reaction was carried out by “Dynabeads” Protein G (Invitrogen), previously incubated for 2 h at room temperature. Immunocomplexes are eluted from the beads with an Elution Buffer [31]. The following RNA clean-up and concentration are performed using Kit “RNA Clean & Concentrator −5” (Zymo Research, EuroClone), according to manufacturer’s instructions.

### Analysis of WTAP expression in breast cancer datasets

Analysis of WTAP expression levels (mRNA and protein) across all cancer types was performed interrogating GEPIA (http://gepia.cancer-pku.cn/) web resource.

The TCGA BRCA cohort, containing data from 24 tumor samples profiled by RNAseq, whole-exome sequencing (WES) and 98 tumor samples profiled for protein levels (mass spectrometry by CPTAC), was used for the correlation of WTAP expression and survival outcome. Comparisons of WTAP expression, at mRNA and protein levels, between breast normal and cancer samples, as well as between breast normal tissue and the various breast cancer subtypes were carried out by interrogating the UALCAN web resource (http://ualcan.path.uab.edu/cgi-bin/ualcan-res-prot.pl).

### Statistical analysis

Time to event endpoints (PFS, DSS and OS) were estimated using the Kaplan-Meier product-limit method, using the log-rank test for subgroup comparison. All results were reported as means ± SDs for at least 3 independent experiments. Statistical significance was analyzed by Student’s *t*-test and expressed as a *P* value. R (“survival” and “survminer” packages) were used for statistical analyses. The level of significance was defined as *p* < 0.05.

### Supplementary information

Supplementary Fig. Legends

Supplementary Fig. 1

Supplementary Fig. 2

Supplementary Fig. 3

Supplementary Fig. 4

Supplementary Fig. 5

Supplementary tables

## Data Availability

Sequencing data were deposited into the Gene Expression Omnibus database under accession number GSE240616 and are available at the following URL: https://www.ncbi.nlm.nih.gov/geo/query/acc.cgi?acc=GSE240616.
