# Supplementary material for: The lncRNAMALAT1-WTAP axis: a novel layer of EMT regulation in hypoxic triple-negative breast cancer
Source: Cell Death Discov. 2024 Jun 11;10:276. doi: 10.1038/s41420-024-02058-4 (PMC11166650; doi:10.1038/s41420-024-02058-4)
Supplement: Supplementary file 1 — Supplementary Fig. Legends [file 41420_2024_2058_MOESM1_ESM.docx]

Supplementary Material

**Supplementary Figure 1. (A)** Immunofluorescence staining of WTAP protein in MDA-MB-231 after transfection of siRNA to silence MALAT1 (si-MALAT1) or control siRNA (si-NC) for 72h (48h of which in hypoxia). Nuclei were stained with DAPI. **(B)** RIP assay to evaluate the enrichment of WTAP in samples immunoprecipitated with anti-eIF4BB antibody or IgG in control or MALAT1 depleted MDA-MB-231 hypoxic cells. p-value has been calculated by unpaired, two-tailed Student’s t test. **(C)** Box plot showing the expression of WTAP mRNA and protein in TCGA and CPTAC datasets of Lung Adenocarcinoma p***<0.0005. **(D)** Kaplan-Meier showing disease free survival (DFS) between the two groups: High WTAP protein expression (N=13 patients) and Low WTAP protein expression (N=77 patients) in TNBC patients (p=0.022). **(E)** RT-qPCR analysis of respectively HIF1α and HIF1β in MDA-MB-231 cells grown in hypoxia transfected with a siRNA to silence MALAT1 (si-MALAT1) or control siRNA (si-NC) for 72h. (N = 3 independent biological replicates).

**Supplementary Figure 2.**  **(A)** PI staining indicating percentage of live cells transfected with siRNA directed to WTAP (si-WTAP) and MALAT1 (si-MALAT1) or control (si-NC) after 48h of hypoxia. 24h staining was used as control **(B)** CFSE staining peaks showing proliferation in MDA-MB-231 cells treated as in A. **(C-F)** RT-qPCR analysis of N-Cadherin and Vimentin showing relative enrichment after m6A precipitation of hypoxic MDA-MB-231. p-value has been calculated by unpaired, two-tailed Student’s t test. **(D-G)** RT-qPCR analysis of N-Cadherin and Vimentin in m6A immunoprecipitated MDA-MB-231 upon silencing of WTAP (si-WTAP) or control siRNA (si-NC) under normoxia for 72h. IgG were used as control. **(E-H)** RT-qPCR analysis of N-Cadherin and Vimentin transcripts in cells treated as in C-F.

**Supplementary Figure 3. (A)** Western blot analysis of WTAP in MDA-MB-468 cells transfected with siRNA directed to MALAT1 (si-MALAT1) or control siRNA (si-NC) for 72h. GAPDH staining was used as loading control. (**B-C**) Representative Western blot analysis of WTAP protein in MDA-MB-468 and MDA-MB-231 after transfection of siRNA to silence MALAT1 (si-MALAT1) or control siRNA (si-NC) grown in hypoxia. (N = 3 independent biological replicates). GAPDH staining was used as loading control.

**Supplementary Figure 4. (A)**  Western blot analysis of HIF1α and WTAP in MDA-MB-231 cells transfected with siRNA directed to WTAP (si-WTAP) (right) or control siRNA (si-NC) for 72h (in hypoxia). GAPDH staining was used as loading control. **(B)** Western blot analysis of HIF1α and WTAP in MDA-MB-231 cells transfected with siRNA directed to MALAT1 (si-MALAT1) or control siRNA (si-NC) for 72h (in hypoxia). GAPDH staining was used as loading control.

**Supplementary Figure 5. (A)** Representative Western blot analysis of N-Cadherin, Vimentin and WTAP proteins in MDA-MB-231 after transfection of siRNA to silence WTAP (si-WTAP) or control siRNA (si-NC) for 72h (48h of which in hypoxia) GAPDH was used as a loading control. **(B)** Representative Western blot analysis of N-Cadherin, Vimentin and WTAP proteins in MDA-MB-468 after transfection of siRNA to silence WTAP (si-WTAP) or control siRNA (si-NC) for 72h (48h of which in hypoxia). **(C)** Western blot analysis of N-Cadherin, Vimentin and WTAP in MDA-MB-231 cells transfected with siRNA directed to MALAT1 (si-MALAT1) or control siRNA (si-NC) for 72h (48h of hypoxia). B-ACTIN staining was used as loading control.
