# Supplementary figures and images for: The lncRNAMALAT1-WTAP axis: a novel layer of EMT regulation in hypoxic triple-negative breast cancer
Source: Cell Death Discov. 2024 Jun 11;10:276. doi: 10.1038/s41420-024-02058-4 (PMC11166650; doi:10.1038/s41420-024-02058-4)

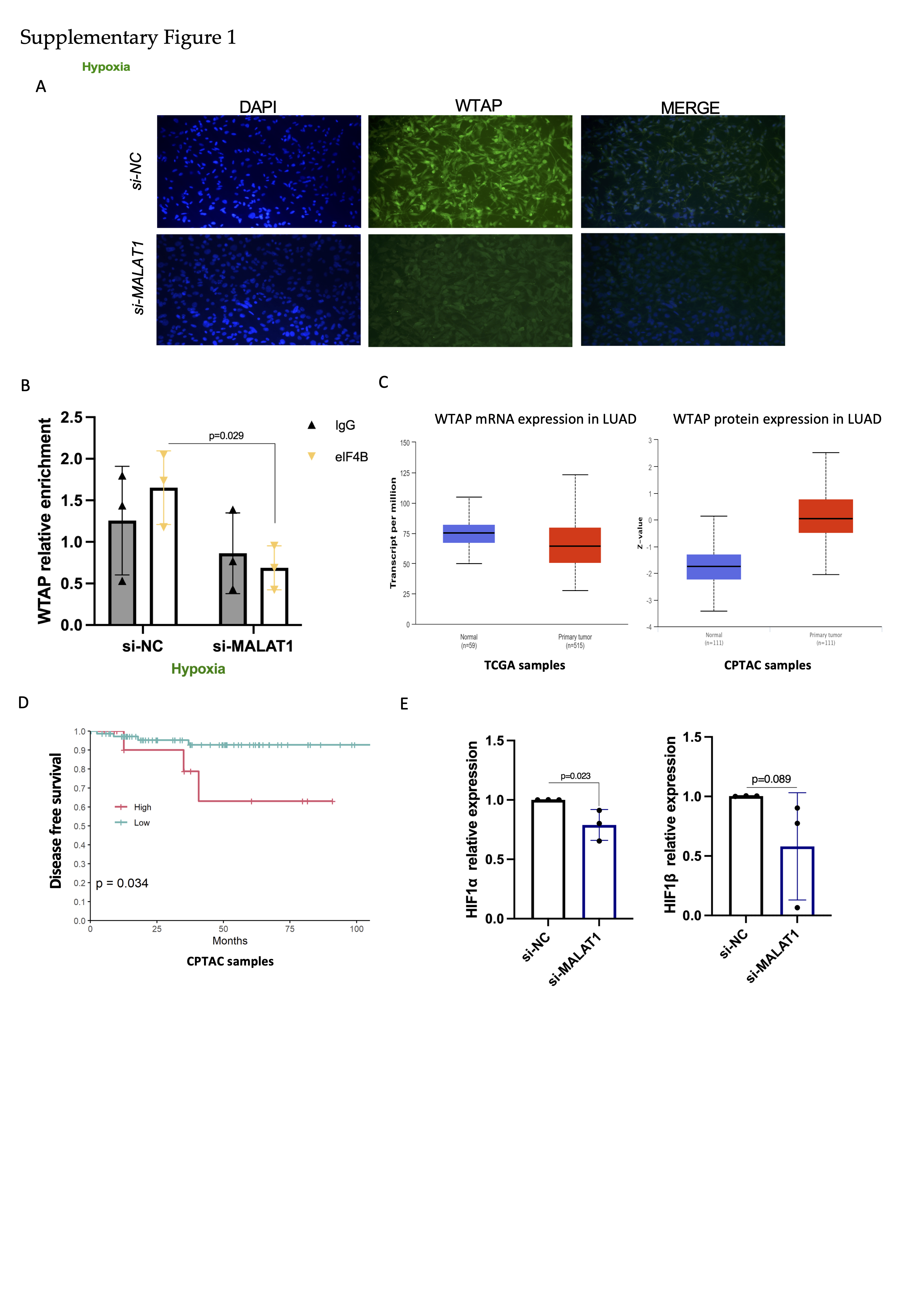

Supplement: Supplementary file 2 — Supplementary Fig. 1 [file 41420_2024_2058_MOESM2_ESM.tif]

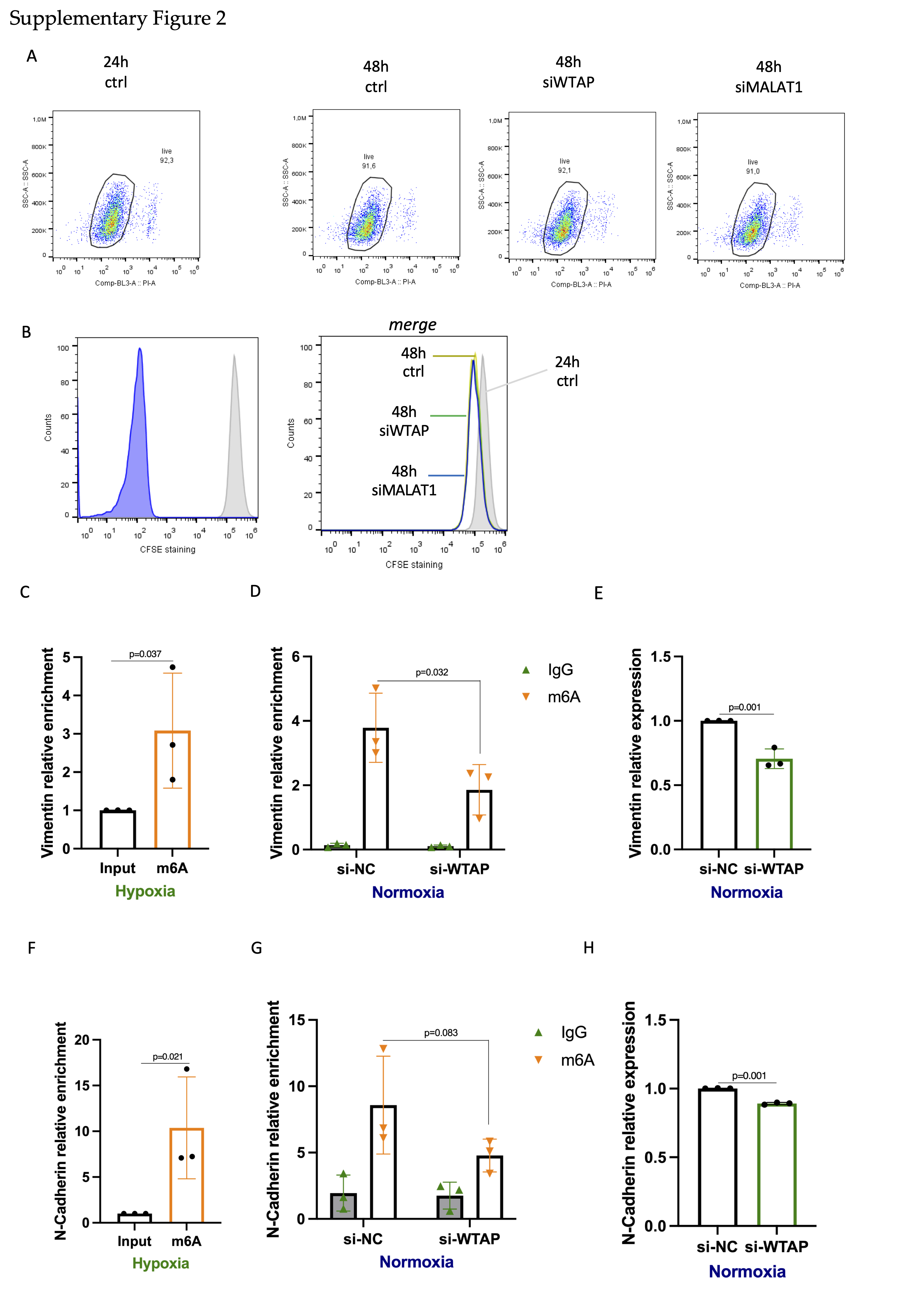

Supplement: Supplementary file 3 — Supplementary Fig. 2 [file 41420_2024_2058_MOESM3_ESM.tif]

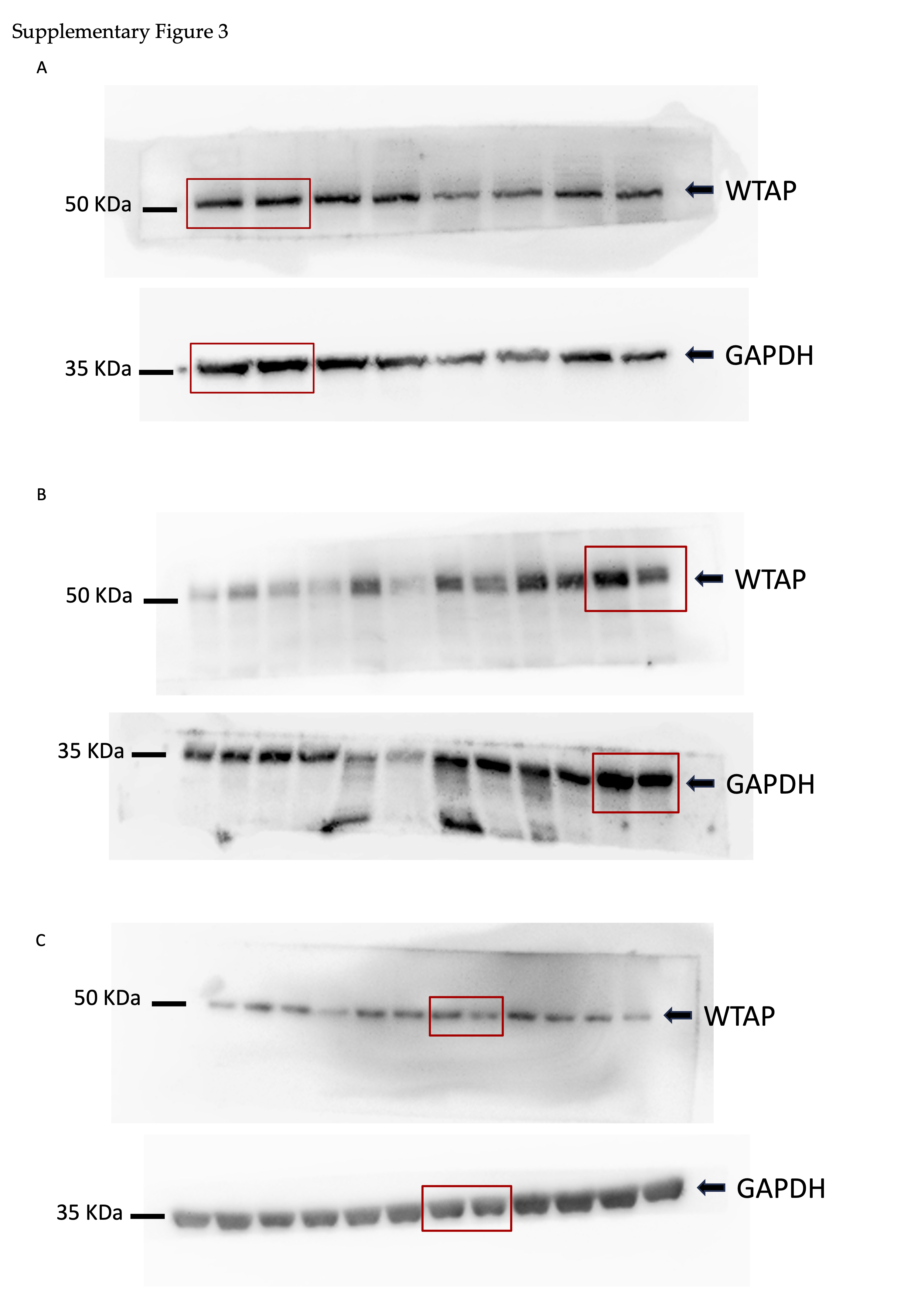

Supplement: Supplementary file 4 — Supplementary Fig. 3 [file 41420_2024_2058_MOESM4_ESM.tif]

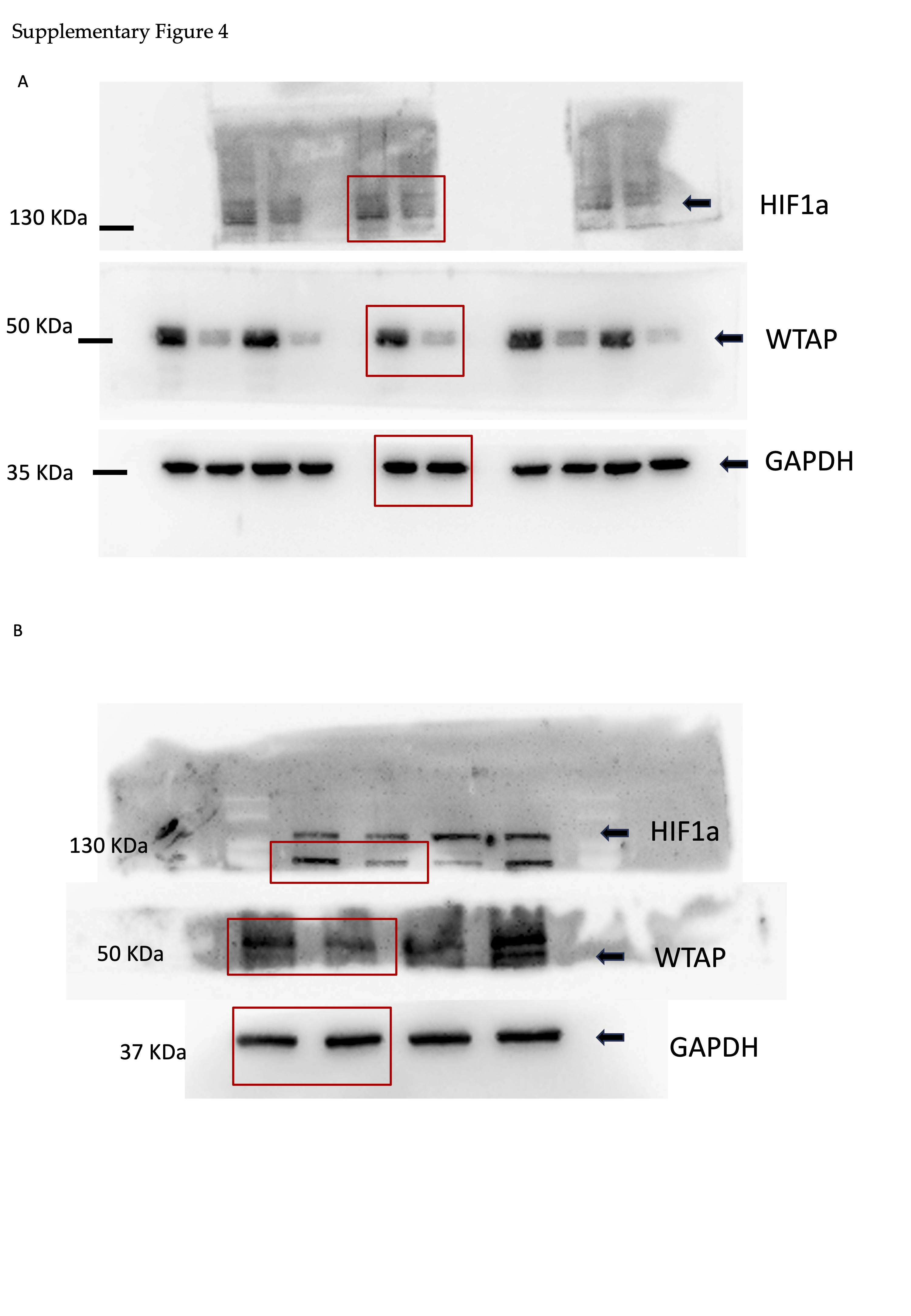

Supplement: Supplementary file 5 — Supplementary Fig. 4 [file 41420_2024_2058_MOESM5_ESM.tif]

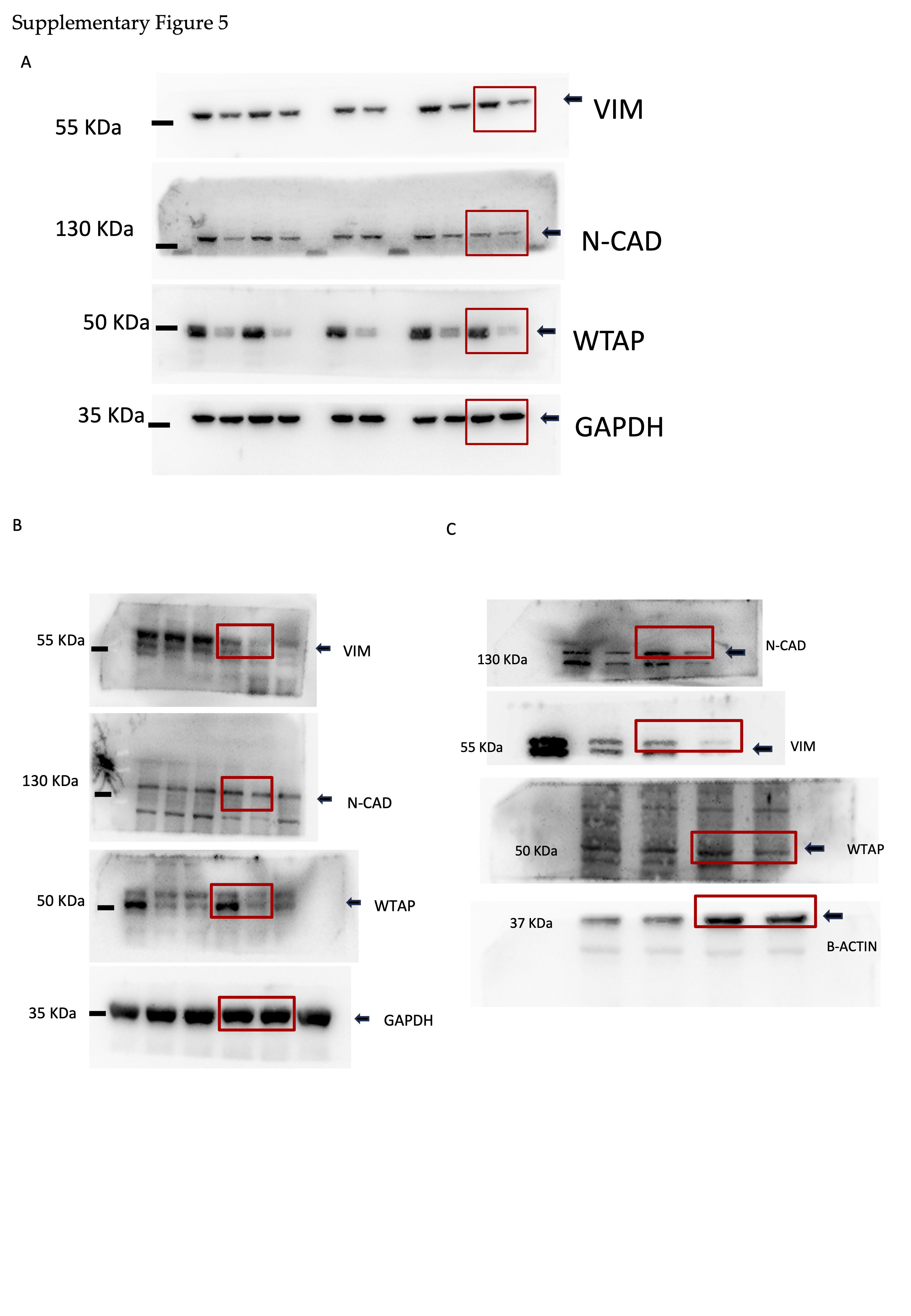

Supplement: Supplementary file 6 — Supplementary Fig. 5 [file 41420_2024_2058_MOESM6_ESM.tif]
