# Supplementary material for: The lncRNAMALAT1-WTAP axis: a novel layer of EMT regulation in hypoxic triple-negative breast cancer
Source: Cell Death Discov. 2024 Jun 11;10:276. doi: 10.1038/s41420-024-02058-4 (PMC11166650; doi:10.1038/s41420-024-02058-4)
Supplement: Supplementary file 7 — Supplementary tables [file 41420_2024_2058_MOESM7_ESM.docx]

**Supplementary Table 1**

| **List EMT genes** | |
| --- | --- |
| CDH1 | SNAI2 |
| VIM | SOX10 |
| CLDN3 | TWIST1 |
| CLDN4 | TWIST2 |
| CLDN7 | ZEB1 |
| DSP | ZEB2 |
| FN1 | MMP2 |
| FOXC2 | MMP3 |
| SNAI1 |  |

**Supplementary Table 2**

| **siRNA** | **Sequence** |
| --- | --- |
| MALAT1#1  (IdThs.Ri. MALAT1.13.1) | For 5’-GCUCCUUGGUGAAUUGAUAAGUAAA-3’ |
|  | Rev 5’-UUUACUUAUCAAUUCACCAAGGAGCUG-3’ |
| NC1 (IdT) | Cat n° 51011403 |

| **siRNA** | **Sequence** |
| --- | --- |
| WTAP#1  (IdThs.Ri. WTAP.13.1) | For 5’- AGAAGGUUCGAUUGAGUGAAACAGA -3’ |
|  | Rev 5’- UCUGUUUCACUCAAUCGAACCUUCUUG-3’ |
| NC1 (IdT) | Cat n° 51011403 |

**Supplementary Table 3**

| **Primer rtPCR** | **FW** | **RV** |
| --- | --- | --- |
| WTAP | GCCTGGAAGTTTACGCCTGA | TTGTGCAATACGTCCCTGGG |
| VIMENTIN | GGACCAGCTAACCAACGACA | GGGTGTTTTCGGCTTCCTCT |
| N-CADHERIN | TGGACCGAGAATCACCAAATGT | ACACTTGAGGGGCATTGTCA |
| HIF1a | ATCTGCAGGTCCCCATTCAA | ATGTGTGTGTGTCGTGTGTG |
| HIF1β | CAAGCCCCTTGAGAAGTCAG | GAGGGGCTAGGGCACTATTC |
| MALAT1 | GGGAAGGCGAAGAAAAGAAT | TGCCCTTAGCTTTTTGTTTCC |
| GAPDH | GAGTCAACGGATTTGGTCGT | GACAAGCTTCCCGTTCTCAG |
| RPL19 | CGGAAGGGCAGGCACAT | GGCGCAAAATCCTCATTCTC |
| RNU2 | CTCGGCCTTTTGGCTAAGAT | TATTCCATCTCCCTGCTCCA |

**Supplementary Table 4**

| **Pull-down** | **Sequence** |
| --- | --- |
| Bio_MALAT1_1 | GCTTCTGCGTTGCTAAAATG/3BioTEG/ |
| Bio_MALAT1_2 | GCGAGGCGTATTTATAGACG/3BioTEG/ |
| Bio_MALAT1_3 | ACGCTAAGCAATATCTTAGT/3BioTEG/ |
| Bio_MALAT1_4 | TAAGCTGTTTAAGTCACCTT/3BioTEG/ |
| Bio_MALAT1_5 | GGTCTTTTAATCACCTTCGG/3BioTEG/ |
| Bio_MALAT1_6  Bio_MALAT1_7 | TCTTTCCA TTTTCGTCTGCG/3BioTEG/  GCCCTTAGCTTTTTGTTTCC/3BioTEG/ |
| Bio_MALAT1_8 | TCTGGTCTACGTAAACACCC/3BioTEG/ |
| Bio_MALAT1_9 | CTGGGTCAGCTGTCAATTAA/3BioTEG/ |
| Bio_MALAT1_10 | TTGCTCCTCAGTCCTAGCTT/3BioTEG/ |
| Bio_MALAT1_11 | TGGAATCCTTTTTCCTAGCT/3BioTEG/ |
| Bio_MALAT1_12 | TGGTGGTCTGATTATCCTGA/3BioTEG/ |
| Bio_MALAT1_13 | ATTAATGCACTGGTACACCC/3BioTEG/ |
| Bio_MALAT1_14 | CATCAAGGCACTGATCACTT/3BioTEG/ |
| Bio_MALAT1_15 | ATGCATTCTAATAGCAGCGG/3BioTEG/ |
| Bio_MALAT1_16 | CTTCAAGATTCCTTCGGAT/3BioTEG/ |
| Bio_LacZ_1 | GTCATATGCATAAAGCGTTG/3BioTEG/ |
| Bio_LacZ_2 | TTAACGCCGCAGTGGTAGAA/3BioTEG/ |
